# Trait anxiety and the neural efficiency of manipulation in working memory

**DOI:** 10.3758/s13415-012-0100-3

**Published:** 2012-05-29

**Authors:** Ulrike Basten, Christine Stelzel, Christian J. Fiebach

**Affiliations:** 1 Department of Psychology, Goethe University, Postfach 11 19 32, Fach 128, 60054 Frankfurt am Main, Germany; 2 Department of Neuroradiology, University of Heidelberg, INF 400, 69120 Heidelberg, Germany; 3 Department of Psychiatry and Psychotherapy, Charité Universitätsmedizin, Charitéplatz 1, 10117 Berlin, Germany; 4 IDeA Center for Individual Development and Adaptive Education, Mertonstr. 17, 60325 Frankfurt am Main, Germany; 5 Centre for Cognition, Donders Institute for Brain, Cognition and Behaviour, Radboud University Nijmegen, Montessorilaan 3, 6525 HR Nijmegen, The Netherlands

**Keywords:** Anxiety, Attention, Cognitive control, Functional connectivity, Personality, Working memory

## Abstract

The present study investigates the effects of trait anxiety on the neural efficiency of working memory component functions (manipulation vs. maintenance) in the absence of threat-related stimuli. For the manipulation of affectively neutral verbal information held in working memory, high- and low-anxious individuals (*N* = 46) did not differ in their behavioral performance, yet trait anxiety was positively related to the neural effort expended on task processing, as measured by BOLD signal changes in fMRI. Higher levels of anxiety were associated with stronger activation in two regions implicated in the goal-directed control of attention—that is, right dorsolateral prefrontal cortex (DLPFC) and left inferior frontal sulcus—and with stronger deactivation in a region assigned to the brain’s default-mode network—that is, rostral–ventral anterior cingulate cortex. Furthermore, anxiety was associated with a stronger functional coupling of right DLPFC with ventrolateral prefrontal cortex. We interpret our findings as reflecting reduced processing efficiency in high-anxious individuals and point out the need to consider measures of functional integration in addition to measures of regional activation strength when investigating individual differences in neural efficiency. With respect to the functions of working memory, we conclude that anxiety specifically impairs the processing efficiency of (control-demanding) manipulation processes (as opposed to mere maintenance). Notably, this study contributes to an accumulating body of evidence showing that anxiety also affects cognitive processing in the absence of threat-related stimuli.

**Electronic supplementary material:**

The online version of this article (doi:10.3758/s13415-012-0100-3) contains supplementary material, which is available to authorized users.

Individual differences in trait anxiety have been associated with differences in cognitive functioning (Bishop, 2007; Eysenck, Derakshan, Santos, & Calvo, 2007; Mathews & Mackintosh, 1998). The present study investigates the effects of trait anxiety on the neural efficiency of cognitive processing in the absence of threat-related stimuli. In a previous study, we showed that for inhibitory control in the Stroop task, trait anxiety was associated with reduced neural efficiency, in terms of weaker functional coupling within a network of task-relevant brain regions and increased activation of a prefrontal control region—that is, the right dorsolateral prefrontal cortex (DLPFC; Basten, Stelzel, & Fiebach, 2011). Here, we tested whether or not trait anxiety also modulates efficiency in terms of both brain activation and functional coupling during the manipulation of working memory contents.

It has been postulated that anxiety impairs cognitive processing due to an impairment of the goal-directed control of attention. This assumption stems from research on the processing of threat-related information by anxious individuals (Mathews & Mackintosh, 1998; Windmann, 1998) and has also been applied to cognitive processing in the absence of threat-related information (Bishop, 2007; Eysenck et al., 2007). On the basis of the common assumption that the goal-directed control of attention is impaired in trait-anxious individuals, different predictions have been put forward concerning the neural correlates of this impairment. While Bishop (2007, 2009) expected high-anxious individuals to show weaker activation of brain regions supporting cognitive control than would low-anxious individuals, Eysenck and colleagues (2007; Eysenck & Derakshan, 2010) argued that high-anxious individuals should show stronger activation of these brain regions, reflecting an attempt to gain control by compensatory increases in neural effort expended on task processing, and thereby to maintain performance at a high level. With our fMRI studies, we aimed at testing the predictions put forward by Eysenck et al. (2007) in their attentional control theory.

Attentional control theory (Eysenck et al., 2007) distinguishes between *performance effectiveness* und *processing efficiency*. While *performance effectiveness* refers to the quality of performance, as usually assessed by performance accuracy, *processing efficiency* relates the observed effectiveness to the *effort* invested in task processing: the higher the effort expended to reach a given level of performance effectiveness, the lower the efficiency of processing. The central prediction of attentional control theory is that anxiety impairs the efficiency of processing more than it impairs the effectiveness of performance. It is assumed that high-anxious individuals expend compensatory effort on task processing in order to make up for their poorer attentional control. On the one hand, this may enable them to keep a level of performance comparable to that of low-anxious individuals, but on the other hand, it renders their processing less efficient.

Support for the assumptions of attentional control theory has come from behavioral studies that have used performance accuracy to assess effectiveness and response times to assess effort and efficiency (Ansari & Derakshan, 2010; Ansari, Derakshan, & Richards, 2008; Derakshan, Ansari, Hansard, Shoker, & Eysenck, 2009; Derakshan, Smyth, & Eysenck, 2009). These studies, however, have been criticized for relying on an indirect measure of effort/efficiency that, furthermore, is difficult to disentangle from the behavioral measure used to assess effectiveness (Ansari & Derakshan, 2011a; Derakshan & Eysenck, 2009), as both response times and accuracy measure the outcome of processing rather than the processing itself. A measure more directly reflecting the effort expended on processing would result from the assessment of brain activity during task processing. In particular, the blood-oxygenation-level-dependent (BOLD) signal measured with functional magnetic resonance imaging (fMRI) is suitable for assessing effort, as the hemodynamic response reflected in this signal is related to neural activity (Logothetis, Pauls, Augath, Trinath, & Oeltermann, 2001; Logothetis & Wandell, 2004) and typically increases with cognitive effort (Braver et al., 1997; Jonides et al., 1997; Manoach et al., 1997; Rypma, Prabhakaran, Desmond, Glover, & Gabrieli, 1999)—constrained by physiologically characterized upper limits (Callicott et al., 1999; Linden et al., 2003; Todd & Marois, 2005).

The first studies using fMRI and electroencephalography (EEG) to investigate the effects of anxiety on brain activation during affectively neutral tasks focused on inhibitory control (Ansari & Derakshan, 2011b; Basten et al., 2011; Bishop, 2009) and task switching (Ansari & Derakshan, 2011a). Some of these studies found stronger, supposedly compensatory, activation in high- as compared to low-anxious participants, along with comparable levels of performance accuracy (Ansari & Derakshan, 2011a) or lower accuracy in high-anxious individuals (Basten et al., 2011)—both patterns (investing more and achieving the same or less) that implicate reduced neural efficiency in high-anxious individuals. Notably, the effect reported in Basten et al. also held when statistically controlling for variation in performance accuracy. Other studies found weaker, supposedly insufficient, activation for the high-anxious, along with slower performance (Ansari & Derakshan, 2011b; Bishop, 2009)—a pattern (investing less and achieving less) not unequivocally interpretable in terms of neural efficiency. So far, only a single fMRI study has investigated the effects of trait anxiety on brain activation during working memory processing. Fales et al. (2008) reported stronger transient activation of cognitive-control regions for high- relative to low-anxious participants during a verbal three-back task, while performance levels did not differ between the two groups. This finding supports the predictions of attentional control theory for the domain of working memory, as the fact that the high-anxious participants invested more neural effort for a comparable level of performance renders their processing less efficient. Yet the *n*-back task chosen by Fales et al. did not allow for specifying which exact component function of working memory was affected by anxiety. With the present study, we aimed at extending the findings of Fales et al. (a) by disentangling whether the effects of anxiety on the neural correlates of working memory are attributable to a specific component function of working memory—that is, to manipulation versus maintenance (see Baddeley, 2003)—and (b) by investigating whether, for working memory, anxiety modulates only the strength of activation in cognitive-control regions, or also the functional coupling of distributed task-relevant networks (like we found for the Stroop task in our previous study; see Basten et al., 2011).

For our investigation, we chose a delayed-response working memory task that allowed us to differentiate between the two component functions defining working memory—that is, maintenance and manipulation (Baddeley, 2003). Performance effectiveness was equated with performance accuracy, the effort invested in task processing was defined as task-related changes in BOLD signal, and processing efficiency was determined by relating accuracy to BOLD signal changes (in formal terms, effectiveness = accuracy, effort = brain activation, and efficiency = accuracy / brain activation). Note that generally when effectiveness (accuracy) does not covary with anxiety, the effects of anxiety on efficiency are simply determined by differences in effort (brain activation).

We hypothesized that anxiety would be negatively correlated with neural efficiency, specifically during working memory manipulation. Only when attentional control requirements come into play (as for the manipulation of working memory contents, as opposed to mere maintenance; see Baddeley, 2003) is anxiety expected to result in compensatory increases in effort and—not necessarily, but potentially also—in an impairment of performance (Eysenck et al., 2007). More specifically, for high- as compared to low-anxious individuals, we predicted that we would find compensatory increases in neural effort in regions of the brain that are known to support cognitive control and executive processes in the context of working memory manipulation. In our analyses, we focused on the bilateral DLPFC (Brodmann’s areas [BAs] 46 and 9). These regions have most consistently been implicated in executive-control processes, in general (Duncan & Owen, 2000; Miller & Cohen, 2001; Smith & Jonides, 1999), as well as in the manipulation (as opposed to the maintenance) of working memory contents, in particular (D’Esposito, Postle, Ballard, & Lease, 1999; D’Esposito, Postle, & Rypma, 2000). So as not to miss any effects of anxiety on brain activation outside our region of interest, we will also report the results from whole-brain analyses. Finally, we predicted that anxiety would affect not only the strength of activation in DLPFC, but also the functional coupling of the DLPFC with other task-related regions.

Even though in our sample anxiety was not significantly associated with a behavioral outcome, we decided to statistically control for subtle variation in performance accuracy. In addition, we controlled for variation in psychometric intelligence. As differences in cognitive ability have previously been associated with differences in neural effort and—consequently—in efficiency (Neubauer & Fink, 2009a), we considered it important to assure that differences in neural measures that were attributed to anxiety could not instead be explained by variation in intelligence. However, like performance, intelligence was not significantly related to anxiety. Thus, statistically controlling for the two variables by multiple regression procedures did not substantially influence the anxiety predictor. Note, finally, that the delayed-response task that we used for the present study could be split into different task periods addressing different cognitive functions—that is, encoding, delay, and retrieval period (D’Esposito et al., 2000). For the present research question, our main focus was on the delay period of the task, as only this task period allowed for a comparison of manipulation-related neural activity with maintenance-related activity. To provide a full picture of the neural processes associated with the task as a whole, activation and the effects of anxiety for the encoding and retrieval periods are reported in the supplementary materials.

## Materials and method

### Participants

The present study was conducted with 46 healthy volunteers who had previously participated in our study on the effects of anxiety on inhibitory control in the Stroop task (Basten et al., 2011). All were students of the University of Heidelberg, were right-handed, and had normal or corrected-to-normal vision, no structural brain abnormalities, and no history of psychiatric or neurological diseases, according to self-report in a telephone interview. Informed consent was obtained in conformity with the protocol approved by the local ethics committee, and the participants were paid for participation in the study. Of the 46 participants, 22 were female and 24 were male, and their ages ranged from 19 to 27 years (*M* = 22.3, *SD* = 2.0). Trait anxiety was assessed with the State–Trait Anxiety Inventory (STAI: Spielberger, Gorsuch, & Lushene, 1970; German: Laux, Glanzmann, Schaffner, & Spielberger, 1981) approximately 6 weeks prior to the study. The raw scores on this measure ranged from 24 to 46 (*M* = 33.3, *SD* = 5.7), which is comparable to the values of a normative German sample of similar age and education (*M* = 34.7, *SD* = 8.4; Laux et al., 1981). For the analysis of variance of the behavioral data and for the illustration of the imaging results in bar plots (see Figs. 2d and 3c below), the sample was median-split into a low-anxious and a high-anxious group, who differed significantly in trait anxiety scores (low-anxious *M* = 28.6, high-anxious *M* = 38.1), *t*(44) = 10.3, *p <* .001, but not in intelligence, as assessed using the Advanced Progressive Matrices (APM: Raven, Raven, & Court, 1998), *t*(44) = 0.70, *p* = .50. Furthermore, trait anxiety did not significantly differ between men (*M* = 33.0, *SD* = 6.2) and women (*M* = 33.7, *SD* = 5.2), *t*(44) = 0.389, *p* = .70. Accordingly, there was no significant difference in the frequency of males versus females in the groups of high-anxious (11 vs. 12) and low-anxious (13 vs. 10) individuals [χ^2^(1) = 0.348, *p* = .56].

### Experimental procedure

**Fig. 1:**
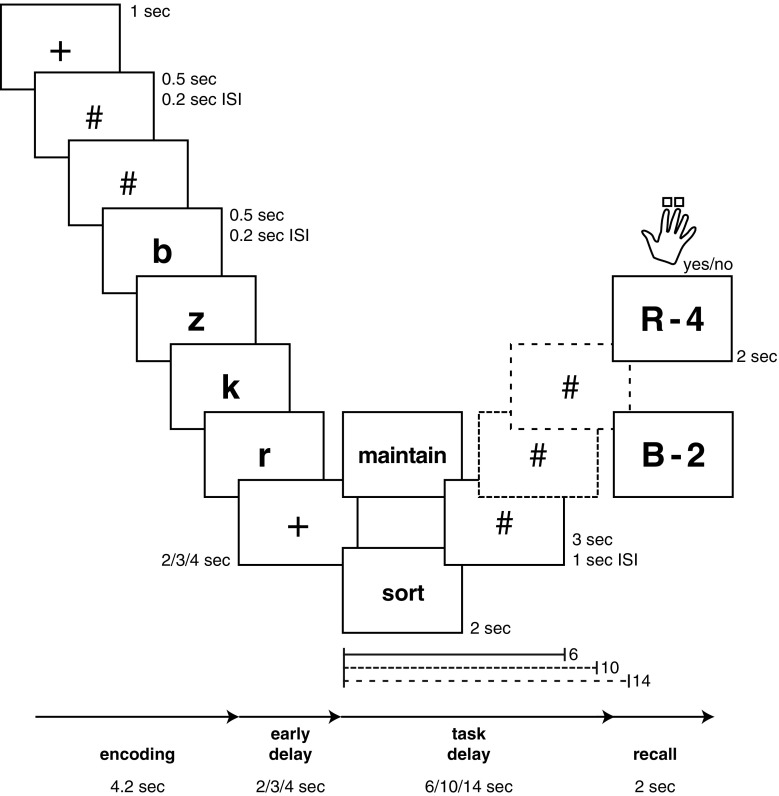
Schematic of the working memory manipulation task. The encoding period comprised the presentation of two hash keys and four letter stimuli. The task delay period comprised the presentation of a verbal task cue followed by one, two, or three hash keys. ISI, interstimulus interval

The participants performed a modified delayed-response task (Fig. 1; D’Esposito et al., 1999; Postle, Berger, & D’Esposito, 1999) including a maintenance and a manipulation condition. The task consisted of three phases: encoding, delay (separated into an early delay period and a task delay period), and recall. In the encoding phase, four sequentially presented letters had to be encoded into working memory. In the early delay phase, participants maintained the encoded set of letters in memory. In the task delay phase, a written cue indicated which task to perform on the four letters. In the maintenance condition (cued by the word “maintain,” *merke*, in German), the participants continued to maintain the letters in the order of presentation (upper stream in Fig. 1). In the manipulation condition (cued by the word “sort,” *sortiere*, in German), the participants mentally rearranged the letters into alphabetical order (lower stream in Fig. 1). In neither of the two conditions were new letters presented during the task delay phase. Instead, the participants saw hash keys (#) that served as placeholders to ensure perceptual equivalence with conditions not considered in the present analyses. In the recall phase, a probe stimulus, consisting of a letter and a number (the latter indicating the position of the letter in the memory set) required retrieval of information from working memory. With the index and middle fingers of their right hands, participants indicated via buttonpress whether or not the given letter was in the indicated position in the four-letter memory set (response options: “yes” or “no”). The probe “R-4,” for instance, asked participants to decide whether or not the letter “R” was in the fourth position in the original (maintenance condition) or alphabetized (manipulation condition) memory set. In the example illustrated in Fig. 1, the correct response would be “yes” for the maintenance condition but “no” for the manipulation condition. Timing information is included in the schematic of the task procedure in Fig. 1.

Preceding each trial, a fixation cross was presented for 1 s. The encoding phase had a fixed duration of 4.2 s. In that time, six stimuli (two placeholders followed by four letter stimuli) were presented for 0.5 s each, with an interstimulus interval (ISI) of 0.2 s. In the early delay phase, a fixation cross was displayed for 2, 3, or 4 s. The following task delay phase, during which participants either manipulated or maintained the list of letters, was of variable length (6, 10, or 14 s), depending on the number of hash keys presented (1, 2, or 3; see the dotted-line screen symbols in Fig. 1). Finally, in the recall phase, the probe stimulus was presented for 2 s. Responses were registered during the presentation of the probe stimulus. Each trial was followed by a variable intertrial interval (ITI) of 1.4 to 6.4 s. Participants were trained on the task prior to the fMRI session, and during training they received feedback whenever a response was incorrect or too slow. The participants were instructed to respond quickly and accurately. During image acquisition in the scanner, they received no feedback on performance. The presentation of the task in the scanner was split into four blocks. Across all blocks, participants completed 24 trials of each condition.

### FMRI acquisition procedures

The MRI data were acquired on a Siemens Trio 3 Tesla MRI scanner equipped with a fast gradient system for echoplanar imaging (EPI) and a birdcage head coil. Participants were stabilized with cushions to restrict their head motion comfortably. A screen, attached to the end of the bore, was visible for participants via a mirror in the head coil. The visual stimuli were presented on a dark background in the center of the screen, using the Presentation software (Neurobehavioral Systems, www.neurobs.com). The functional data were acquired using a T2*-weighted BOLD-sensitive gradient-echo EPI sequence with 32 oblique axial slices of 3-mm thickness, a 1-mm interslice gap, field of view (FOV) 192 mm, matrix size 64 × 64, in-plane resolution 3 × 3 mm, repetition time (TR) 2,500 ms, echo time (TE) 30 ms, and flip angle 80º. Four runs of 440 volumes were acquired. The experiment was set up in an event-related design, jittered to improve the BOLD signal estimation (Dale, 1999). The first six volumes of all four runs were discarded to allow for stable magnetization. For co-registration, a T1-weighed anatomical scan with slice prescription identical to that of the functional scans was acquired. Three-dimensional high-resolution structural data were obtained via a sagittal, T1-weighted magnetization-prepared rapid gradient echo (MP-RAGE) scan with 192 slices of 1-mm thickness, FOV 256 mm, matrix size 256 × 256, in-plane resolution 1 × 1 mm, TR 1,570 ms, TE 2.63 ms, and flip angle 30º.

### fMRI data analyses

All MRI data analyses were carried out using the Statistical Parametric Mapping software package (SPM5; Wellcome Trust Centre for Neuroimaging, London, U.K., www.fil.ion.ucl.ac.uk/spm/software/spm5).

#### Preprocessing

The acquired EPI time series were first slice-time and then motion corrected. All functional volumes were spatially normalized into standard (MNI 152) space according to the normalization parameters resulting from the segmentation of the high-resolution anatomies (voxels resampled to 2 × 2 × 2 mm). Finally, spatial smoothing was applied (8-mm full-width-at-half-maximum Gaussian kernel).

#### Task-related brain activation

To identify regions showing task-related activation for the two conditions of interest (maintenance and manipulation), a general linear model (GLM) accounting for serially autocorrelated data (Friston et al., 1995) was set up for each participant, applying a canonical hemodynamic response function and a temporal high-pass filter (cutoff of 128 s). The functional runs were modeled as separate sessions. The GLMs included separate regressors for all of the experimental conditions (i.e., maintenance, manipulation, and four other conditions not evaluated for the present research question) and task periods (i.e., encoding, early delay, task delay, and recall; see Fig. 1). In addition, the model included covariates of no interest for incorrectly answered trials and the realignment parameters derived from the motion correction in the data preprocessing. The results we subsequently report will focus on the task delay period regressors—that is, the period of the task during which participants actually manipulated (or maintained) information in working memory. The results for the encoding and the retrieval periods are reported in the supplementary materials. For each participant, we defined the following contrasts using the regressors for the respective task periods: maintenance > baseline, manipulation > baseline, and manipulation > maintenance. Results from the single-subject analyses were integrated at the group level in a model treating participants as random effects (Holmes & Friston, 1998). The analyses of task-related brain activations included all voxels in the brain. Given that the main effect of manipulation > maintenance in our large sample produced very strong and extensive activations, we applied a rather conservative statistical threshold to allow for a detailed characterization of peak activations (*p* < .05 familywise error rate [FWE] correction with Gaussian random field theory as implemented in SPM5, at the voxel level, and *p* < .001 FWE corrected, at the cluster level).

#### Effects of anxiety on task-related activation

To test for the effects of trait anxiety on the strength of manipulation-related activation, multiple regression models were set up at the level of the group analyses, including trait anxiety as a predictor for brain activation (manipulation > maintenance). Model 1 included solely trait anxiety (i.e., STAI trait scale scores; see the Participants section) as a predictor. Model 2 additionally included performance accuracy as a predictor. We will focus on this model for the display and interpretation of our results, as it provided the most direct test of our hypotheses concerning processing efficiency—that is, activation strength given a constant level of performance (see the introduction). This model allowed us to test where trait anxiety explained brain activation above and beyond what could also be explained by variation in performance. Model 3 contained psychometric intelligence (APM scores) as an additional predictor to ensure that effects attributed to anxiety could not be explained by differences in cognitive ability. In all three models, statistical tests were conducted for the weight of the trait-anxiety regressor.

As effects of anxiety on brain activation were specifically predicted for the DLPFC (see the introduction), the main analyses of anxiety effects were restricted to an anatomical mask comprising bilateral DLPFC. The mask was generated on the basis of the Talairach Daemon database (TD; Lancaster, Summerlin, Rainey, Freitas, & Fox, 1997; Lancaster et al., 2000) using the WFU PickAtlas toolbox in SPM (Maldjian, Laurienti, Kraft, & Burdette, 2003) and comprised BAs 46 and 9, extended 2 × 1 voxel in each direction, and intersected with the TD template for the middle frontal gyrus. Nonbrain voxels included after dilation were excluded by intersection with the whole-brain mask generated during the group analysis in SPM. The resulting DLPFC mask comprised 2,214 voxels. Group statistical parametric maps for the modulation of task-related activation by trait anxiety within the region of interest are reported after applying an overall threshold of *p* < .05 (corrected for multiple comparisons) constituted by an individual-voxel probability threshold of *p* < .005 [uncorrected, *t*(43) > 2.70], in combination with a minimum-cluster-size threshold of *k* > 28 voxels, as determined via Monte Carlo simulation using the AFNI routine AlphaSim (Ward, 2000; cf. Forman et al., 1995). Taking into account the fact that the power to detect between-subjects effects is typically much lower than the power to detect within-subjects effects (Yarkoni, 2009; Yarkoni & Braver, 2010), this approach provides an FWE correction that at the same time ensures sufficient sensitivity for between-groups effects (see also Basten et al., 2011, for a critique of correction methods leading to increased Type II errors—i.e., poor detection of true effects in fMRI research; see Lieberman & Cunningham, 2009). In a second step, the test for anxiety effects on brain activation was extended to consider the whole brain. This analysis was also thresholded at *p* < .05, corrected, with a voxel strength threshold of *p* < .005 [uncorrected, *t*(43) = 2.70] and a cluster size threshold of *k* > 142 voxels (AlphaSim; Ward, 2000).

#### Functional connectivity

To explore the task-related functional connectivity of the DLPFC region showing a modulation of task-related activation by anxiety with distal brain regions, we conducted psychophysiological interaction analyses (PPI; Friston et al., 1997). The procedure, as implemented in SPM5, models the contribution of a seed region to any voxel in the brain by a linear regression model. As the primary seed region, we chose the right DLPFC cluster showing an anxiety effect in the univariate activation analysis within our a-priori-defined region of interest (see the Results section). This cluster fell within the DLPFC cluster activated for working memory manipulation across all participants. In addition to the PPI analysis using the seed within our DLPFC region of interest, we performed two further PPI analyses using as a seed the regions in the left inferior frontal sulcus (IFS) and the rostral-ventral part of the anterior cingulate cortex (rACC) regions identified in the whole-brain analysis of anxiety effects on manipulation-related activity. At the single-subject level, the GLMs contained three regressors: a P regressor, representing the psychological variable (i.e., the task condition, manipulation > maintenance); a Y regressor, representing the physiological variable (i.e., the mean time course of activation in the respective seed region); and a PPI regressor, representing the interaction of the psychological and the physiological regressor. To test for PPI effects across participants at the group level (independent of trait anxiety), the single-subject contrast images testing for an effect of the PPI regressor were entered into a second-level random effects analysis for a *t* test.

To test whether the parameter estimates of the interaction terms could be predicted by trait anxiety, regression models were set up at the group level. As in the analyses testing for anxiety effects on univariate activation strength, three different regression models were set up. Model 1 comprised only trait anxiety as a predictor. In Model 2 we added performance accuracy, and in Model 3 we added a further predictor, intelligence. For all three models, statistical tests were conducted for the effect of the trait-anxiety regressor. Again, the main focus was on Model 2 testing, where anxiety could explain functional connectivity when variation in performance was statistically controlled for. Results are reported for the whole brain, applying the same threshold used for the analysis of anxiety effects on univariate activations—that is, *p* < .05 (corrected), here constituted by a voxel probability threshold of *p* < .005 [uncorrected, *t*(43) = 2.70], in combination with a minimum-cluster-size threshold of *k* > 142 voxels (AlphaSim; Ward, 2000).

#### Offline illustration of brain activation and connectivity estimates

From the regions showing a significant effect of trait anxiety (on regional activation or functional connectivity), individual contrast values were extracted to illustrate the patterns of activation and connectivity as depending on anxiety and task condition. No secondary inferential statistics were done on the plotted data, to avoid problems with nonindependent testing (Poldrack & Mumford, 2009; Vul, Harris, Winkielman, & Pashler, 2009). Scatterplots serve to illustrate that the correlations between the trait-anxiety scores and contrast values were not driven by outliers. Bar plots disentangle the effects of task condition (manipulation vs. maintenance) and anxiety (high vs. low) on the neural measures, by additionally displaying the average activations for the simple effect of maintenance (i.e., maintenance > baseline) in the anxiety-sensitive clusters.

## Results

### Behavioral performance

A significant effect of task condition on recall performance was observed for error rates (maintenance, 6.97 %; manipulation, 14.67 %), *F*(1, 44) = 29.56, *p* < .001, as well as for response times (maintenance, 1,140.42 ms; manipulation, 1,164.89 ms), *F*(1, 44) = 5.412, *p* = .03, suggesting that the manipulation of working memory contents was more difficult than mere maintenance. However, performance was not affected by trait anxiety: Neither for error rates nor for response times were the main effects of anxiety or the interaction effects of anxiety with condition statistically significant (all *p*s > .25).

### Anxiety and regional brain activation

#### Task-related brain activation across all participants

**Fig. 2:**
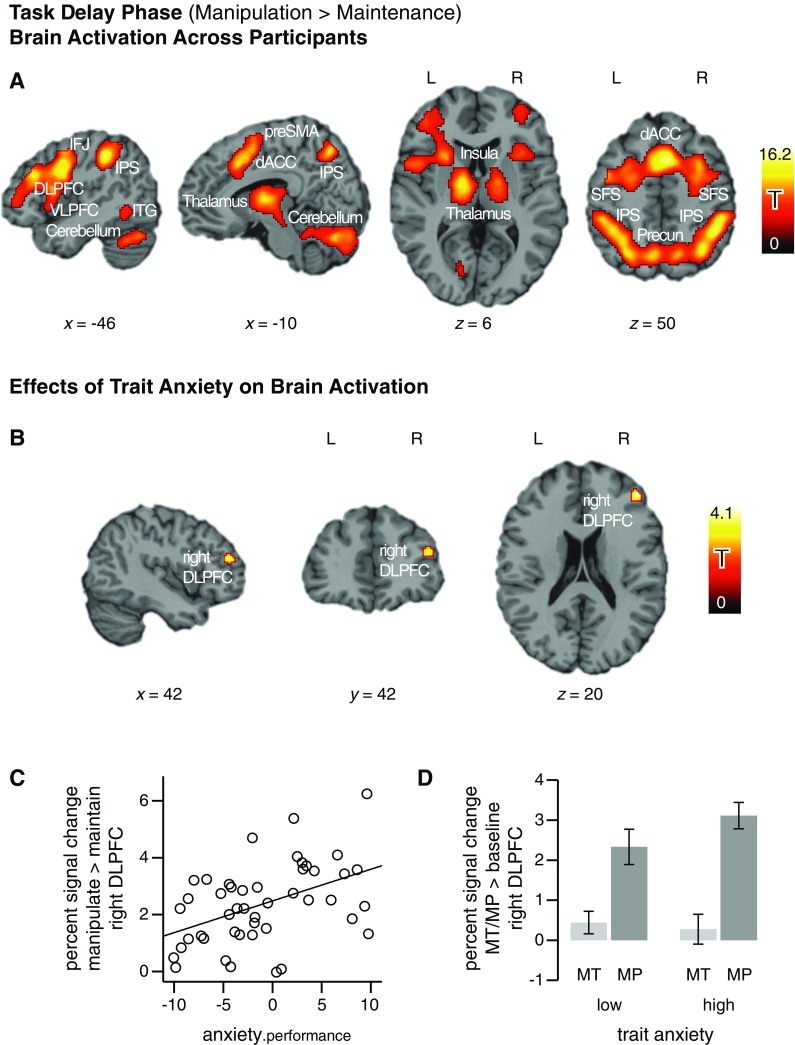
Brain activation during working memory manipulation (manipulation > maintenance). The *x-*, *y-*, and *z*-coordinates refer to the Montreal Neurological Institute template brain included in the SPM5 software package. (**a**) Activation across all participants, illustrated at a voxel-level threshold of *p* < .05 and a cluster-level threshold of *p* < .001, both corrected for familywise error rates. (**b**, **c**, and **d**) Effects of trait anxiety on brain activation during working memory manipulation in the DLPFC region of interest—controlling for (nonsignificant) variations in performance. Trait anxiety predicts BOLD signal strength for an area in the right DLPFC. (**b**) For illustration purposes, statistical parametric maps are shown at a voxel-level threshold of *p* < .01. (**c**, **d**) Percent signal change extracted from the right DLPFC region where anxiety significantly predicted task activation. (**c**) Percent signal change for manipulation > maintenance, plotted against anxiety.performance—that is, the residual of trait anxiety from the regression on behavioral performance. (**d**) Comparison of mean percent signal change for the task (MP: manipulation > zero, dark gray) and the reference condition (MT: maintenance > zero, light gray) by the trait anxiety group (median split). Error bars show the standard errors of the means. dACC, dorsal anterior cingulate cortex; DLPFC, dorsolateral prefrontal cortex; IFJ, inferior frontal junction; IPS, intraparietal sulcus; ITG, inferior temporal gyrus; Precun, Precuneus; preSMA, presupplementary motor area; SFS, superior frontal sulcus; L, left; R, right

During the task delay phase, across all participants—not taking into account differences in trait anxiety—increased activation was observed for the manipulation of working memory contents, as contrasted to mere maintenance, in frontal, parietal, temporal, and subcortical areas of the brain (*p* < .05, corrected; see Fig. 2a; for peak voxel coordinates, *t* values, and cluster sizes, see Table 1). In the lateral frontal cortex, activation comprised bilateral foci in the frontopolar cortex, the DLPFC, the ventrolateral prefrontal cortex (VLPFC, in the left hemisphere comprising the area of Broca), the inferior frontal junction area (IFJ), and the superior frontal sulcus (SFS). In the medial frontal cortex, activation was observed in dorsal ACC and presupplementary motor area (preSMA). Furthermore, activation was significantly increased in the anterior insula. In parietal cortex, an extensive cluster of activation comprised bilateral foci in the intraparietal sulci (IPS) as well as the precuneus, reaching from the lateral cortical surface into the medial wall. Smaller clusters of activation were located in the inferior temporal gyrus (ITG), bilaterally. Subcortical activation was observed bilaterally in the thalamus. Finally, the activation maps showed strong activity increases in superior parts of both cerebellar hemispheres. Brain activation observed for the encoding and the retrieval periods of the task is reported in the supplementary materials (Table S1, Fig. S1).

**Table 1:** Effects of task on brain activation

			MNI				
Brain Region	BA	Hem	*x*	*y*	*z*	*t*_max_	*k*
**Task Delay Phase** (Manipulation > Maintenance) Voxel height threshold *t* = 5.46, Cluster extend threshold: *k* = 25 voxels							
Frontopolar cortex, dorsolateral prefrontal cortex, ventrolateral prefrontal cortex, inferior frontal junction, superior frontal sulcus, dorsal anterior cingulate cortex, presupplementary motor area, anterior insula, thalamus, midbrain	6/8/9/10/32/44/46	L/R	4	12	50	14.78	14372
Frontopolar cortex, dorsolateral prefrontal cortex, inferior frontal junction anterior insula	6/8/9/10/44/46	R	42	38	28	11.80	3570
Intraparietal sulcus, precuneus	7/40	R/L	40	−44	44	16.15	6346
Postcentral gyrus	1	L	−62	−18	24	6.80	44
Cuneus	17	L	−16	−76	6	6.12	38
	17	R	14	−76	10	6.19	34
Inferior temporal gyrus	37	R	56	−50	−12	6.69	32
	37	L	−50	−54	−12	7.79	187
Cerebellum		R/L	28	−68	−28	15.78	7619

BA, approximate Brodmann’s area; Hem, hemisphere; L, left; R, right; MNI, coordinates referring to the Montreal Neurological Institute template brain included in the SPM5 software package; *t*_max_, maximum *t* statistic in the cluster; *k*, cluster size in voxels. Activation is reported for a voxel-level threshold of *p <* .05 and a cluster-level threshold of *p <* .001, both corrected for familywise error rate.

#### Effects of trait anxiety on brain activation during working memory manipulation

Our main analyses concerning the effects of anxiety focused on the bilateral DLPFC, our region of interest for effects of anxiety on neural correlates of attentional-control processes. During the delay period of the task, trait anxiety predicted the strength of manipulation-related activation for a subregion of the right DLPFC (result from Regression Model 1, including only trait anxiety as a predictor; peak MNI coordinates 44, 44, 20, cluster size *k* = 39 voxels, *t*_max_ = 4.20, *p <* .05 corrected for multiple comparisons). In this part of the DLPFC, high-anxious participants showed a stronger increase in brain activation for manipulation as compared to maintenance than did low-anxious individuals. Crucially, trait anxiety explained variation in activation strength that could not be accounted for by variation in behavioral performance (result from Regression Model 2, including trait anxiety and performance accuracy as predictors; MNI coordinates and *p* as for Model 1, cluster size *k* = 36 voxels, *t*_max_ = 4.11; this effect is illustrated in Fig. 2). The scatterplot in Fig. 2c illustrates the positive association between anxiety and brain activation and demonstrates that the effect showing up in the statistic parametric group map was not driven by outliers.

Furthermore, we ensured that the effect of anxiety was specific to the task condition of interest (manipulation) and not attributable to a reverse effect in the reference condition (maintenance). The bar plot in Fig. 2d illustrates that for the reference condition (i.e., for the simple effect of maintenance > baseline), the high- and the low-anxious groups did not differ in their levels of activation [*t*(44) = 0.35, *p* = .73]. In fact, the high-anxious group showed a significantly greater increase from the reference condition (maintenance) to the task condition of interest (manipulation).

When including intelligence in the prediction of brain activation (Regression Model 3), the peak coordinates of the cluster where anxiety contributed significantly in explaining task-related activation remained unchanged, and cluster size changed only slightly, to *k* = 30 voxels (*t*_max_ = 4.91, *p <* .05, corrected). Thus, the incremental contribution of anxiety in explaining activation strength—beyond what could be explained by performance and intelligence—remained significant. No region within the bilateral DLPFC showed the opposite pattern—that is, a negative correlation between anxiety and activation.

**Fig. 3:**
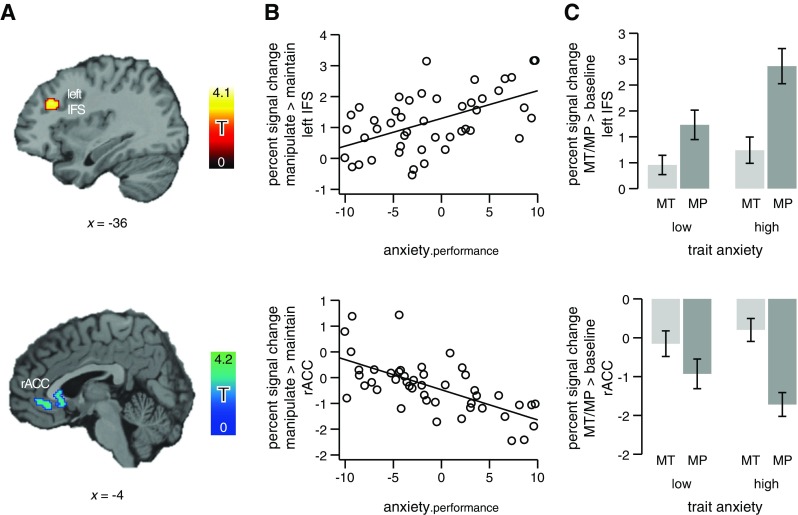
Effects of trait anxiety on brain activation during working memory manipulation in the whole-brain analysis, controlling for (nonsignificant) variation in performance. Trait anxiety predicts BOLD signal strength in the left inferior frontal sulcus (IFS; upper row) and in the rostral–ventral anterior cingulate cortex (rACC; lower row). (**a**) Statistical parametric maps are shown at a voxel-level threshold of *p* < .005. The *x*-coordinates refer to the Montreal Neurological Institute template brain included in the SPM5 software package. (**b**, **c**) Percent signal change extracted from the two regions illustrated in panel A. (**b**) Percent signal change for manipulation > maintenance, plotted against anxiety.performance—that is, the residual of trait anxiety from the regression on behavioral performance. (**c**) Comparison of mean percent signal change for the task (MP: manipulation > zero, dark gray) and the reference condition (MT: maintenance > zero, light gray) by the trait-anxiety group (median split). Error bars show the standard errors of the means

In a second step, we extended our search volume beyond our theoretically derived region of interest to test for effects of anxiety in other parts of the brain. A whole-brain analysis revealed that anxiety predicted manipulation-related activation increases also in three other regions. Here, we focus on the effects of anxiety when controlling for behavioral performance (Regression Model 2). A positive correlation between trait anxiety and task-related activation was observed for a region in the depth of the left IFS and a region in the left brainstem (*p* < .05, corrected; see Table 2A; illustrated for left IFS in Fig. 3a, top). These two regions showed an effect equivalent to that found in the right DLPFC (illustrated for left IFS in Fig. 3b and c, top). The cluster showing an effect in the left IFS was situated rather deep in the sulcus, so that it did not fall within our region of interest defined for bilateral DLPFC.

**Table 2:** Effects of trait anxiety on brain activation (manipulation > maintenance)

				MNI				
Brain Region	BA	Hem	Model	*x*	*y*	*z*	*T*_max_	*k*
**(A) Task Delay Phase** (Positive Correlation) Voxel height threshold *t* = 2.70, Cluster extend threshold: *k* = 142 voxels								
Inferior frontal sulcus	46	L	Model 1:	−32	24	26	3.66	n.s. (111)
			Model 2:	−32	24	26	3.80	145
			Model 3:	−32	24	26	3.57	n.s. (103)
Brainstem (pons, midbrain)		L	Model 1:	−8	−26	−18	3.75	n.s. (115)
			Model 2:	−10	−26	−24	4.03	156
			Model 3:	−16	−28	−34	3.98	n.s. (139)
**(B) Task Delay Phase** (Negative Correlation) Voxel height threshold *t* = 2.70, Cluster extend threshold: *k* = 142 voxels								
Rostral–ventral anterior cingulate cortex			Model 1:	0	18	−4	4.18	600
			Model 2:	6	22	8	4.15	584
			Model 3:	6	22	8	4.20	854

BA, approximate Brodmann’s area; Hem, hemisphere; L, left; R, right; MNI, coordinates referring to the Montreal Neurological Institute template brain included in the SPM5 software package; *t*_max_, maximum *t* statistic in the cluster; *k*, cluster size in voxels; n.s., not significant. Model 1, regression of PPI on trait anxiety. Model 2, regression of PPI on trait anxiety and performance. Model 3, regression of PPI on trait anxiety, performance, and intelligence.

For the third region, in the rACC, we observed a negative correlation between anxiety and brain activation (*p* < .05, corrected; see Table 2B and Fig. 3a, bottom). Plotting the percent signal change for this region (see the bar plot in Fig. 3c, bottom) revealed that the effect was attributable to high-anxious participants showing stronger deactivation for manipulation as contrasted to maintenance than did low-anxious participants. Note that the rACC, a region typically assigned to the task-negative (or default) network (Raichle et al., 2001), also was part of the task-negative network in the present study. It showed deactivation for both task conditions (maintenance and manipulation) as contrasted to baseline (*p* < .05, corrected; for a full description of the task-negative network for the present task, see the supplement, Table S3 and Fig. S3).

For reasons explicated in the introduction, our main interest was in the effects of anxiety on brain activation during the actual manipulation of information in working memory—that is, during the delay period of the task. In the supplementary materials, we provide equivalent analyses for the encoding and retrieval periods (but note that anxiety effects on brain activation during encoding cannot be analyzed separately for manipulation and maintenance conditions, as participants were cued as to the specific task condition after encoding). Whereas during the encoding period trait anxiety did not affect brain activation, during the retrieval period we observed an interaction of task condition and trait anxiety on activation strength, with a focus on parietal regions (see Table S2, Fig. S2).

### Anxiety and functional brain network connectivity

#### Task-related functional connectivity of DLPFC

**Fig. 4:**
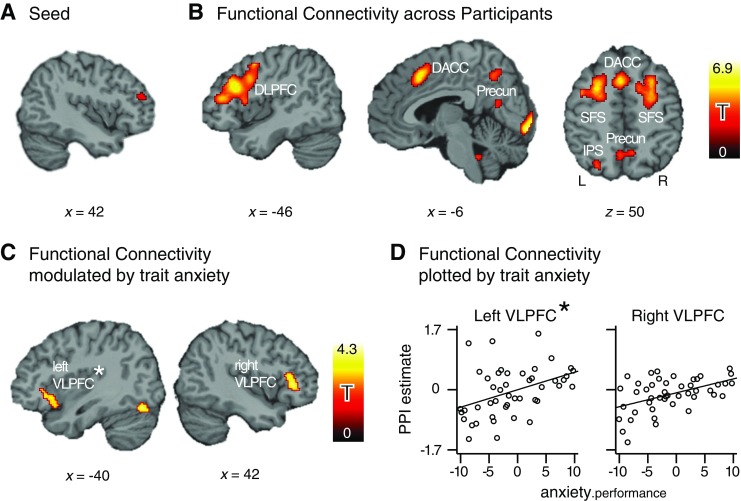
Functional connectivity of the right DLPFC in the working memory manipulation task. The *x-* and *z*-coordinates refer to the Montreal Neurological Institute template brain included in the SPM5 software package, and the statistical parametric maps in panels B and C are shown at a voxel-level threshold of *p* < .005. (**a**) Seed region in right DLPFC. (**b**) Functional connectivity of the right DLPFC across participants, clusters show enhanced coupling with right DLPFC for manipulation > maintenance. (**c**) Positive association of anxiety and functional connectivity, controlling for (nonsignificant) variation in performance. The clusters show stronger PPI with DLPFC in high- as compared to low-anxious participants. (**d**) Individual strength of PPI with DLPFC, plotted against anxiety.performance—that is, the residual of trait anxiety from regression on behavioral performance. The PPI estimates derive from the contrast value of the interaction regressor in the PPI model. The scale of the ordinate given in the plot to the left is valid for both plots. DACC, dorsal anterior cingulate cortex; DLPFC, dorsolateral prefrontal cortex; IPS, intraparietal sulcus; Precun, precuneus; SFS, superior frontal sulcus; VLPFC, ventrolateral prefrontal cortex; L, left; R, right. ^*^Note that seed connectivity with left VLPFC was significantly modulated by anxiety only in the regression model including intelligence

Across participants—not taking into account differences in trait anxiety—a subset of the areas identified as activated during working memory manipulation (see above and in Fig. 2) showed increased functional connectivity with the right DLPFC seed region (Fig. 4a) during the task delay period. For the experimental condition of interest (i.e., manipulation), as contrasted to the control condition (maintenance), activity in the right DLPFC seed region showed enhanced coupling with activity in the left DLPFC (including medial frontal gyrus and the adjacent inferior and superior frontal gyrus), the dorsal ACC, the posterior part of the SFS bilaterally, left IPS, medial parts of the precuneus bilaterally, and superior parts of the right cerebellum (*p* < .05, corrected; see Table 3 and Fig. 4b). Functional connectivity across participants for left IFS and rACC is reported in the supplementary materials (Table S4).

**Table 3:** Functional connectivity of the right DLPFC seed region during working memory manipulation

			MNI				
Brain Region	BA	Hem	*x*	*y*	*z*	*t*_max_	*k*
**Task Delay Phase** (Manipulation > Maintenance) Voxel height threshold *t* = 2.70, Cluster extend threshold: *k* = 142 voxels							
Dorsolateral prefrontal cortex, middle frontal gyrus, inferior frontal gyrus, superior frontal gyrus, dorsal anterior cingulate cortex, superior frontal sulcus	6/8/9/32/44/45/46	L/R	−30	10	58	6.94	4035
Intraparietal sulcus, Precuneus	7/19/39	L	−34	−80	40	4.46	297
Precuneus, Posteriorer cingulate cortex	23/31	L/R	−18	−58	18	4.11	442
Precuneus	7	L/R	−8	−56	46	3.74	149
Occipital lobe	17/18	L/R	−10	−92	−10	6.83	1257
Cerebellum		L/R	4	−42	−34	3.68	164
		R	32	−68	−30	4.26	189

BA, approximate Brodmann’s area; Hem, hemisphere; L, left; R, right; MNI, coordinates referring to the Montreal Neurological Institute template brain included in the SPM5 software package; *t*_max_, maximum *t* statistic in the cluster; *k*, cluster size in voxels.

#### Trait anxiety and functional connectivity

Trait anxiety significantly modulated the functional connectivity of the right DLPFC seed region with the right VLPFC. Higher-anxious individuals showed stronger task-specific increases in functional coupling than did lower-anxious individuals (*p* < .05, corrected; see Table 4: Model 1 results) also when controlling for variation in performance (*p* < .05, corrected; see Table 4: Model 2 results, illustrated for right VLPFC in Fig. 4c). When including intelligence in the regression model, connectivity to the left VLPFC was also significantly modulated by trait anxiety (*p* < .05, corrected; see Table 4: Model 3 results; see also Fig. 4c). In addition to bilateral VLPFC, superior parts of both cerebellar hemispheres showed stronger coupling with the right DLPFC in higher-anxious participants. Finally, no region was identified where higher anxiety would have been associated with weaker coupling to the DLPFC seed region. Focusing on effects that were significant in all three models tested, the additional connectivity analyses for the two seed regions outside the DLPFC region of interest revealed that while left IFS did not show differential connectivity depending on trait anxiety, connectivity to rACC was positively correlated with anxiety for two regions in the rACC directly adjacent to the seed region, extending into orbitofrontal cortex and a region situated around the central sulcus, extending from pre- to postcentral gyrus (for details, see the supplement, Table S5).

**Table 4:** Regions showing increased functional connectivity of the right DLPFC seed region in high-anxious participants during working memory manipulation

				MNI				
Brain Region	BA	Hem	Model	*x*	*y*	*z*	*t*_max_	*k*
**Task Delay Phase** (Manipulation > Maintenance) Voxel height threshold *t* = 2.70, Cluster extend threshold: *k* = 142 voxels								
Ventrolateral prefrontal cortex	44/45/47	R	Model 1:	44	32	2	4.35	221
			Model 2:	44	32	2	4.30	200
			Model 3:	44	32	2	4.12	183
	47	L	Model 1:	−30	8	−16	3.19	n.s. (91)
			Model 2:	−30	8	−16	2.94	n.s. (67)
			Model 3:	−30	8	−16	3.54	154
Superior cerebellum, Occipital lobe, Fusiform gyrus, Lingual gyrus	17/18/19	L	Model 1:	−32	−76	−18	3.96	202
			Model 2:	−32	−76	−18	3.91	339
			Model 3:	−32	−76	−18	3.89	336
Superior cerebellum	18/19	R	Model 1:	20	−78	−20	3.65	240
			Model 2:	20	−78	−20	3.70	240
			Model 3:	20	−78	−20	3.59	358

BA, approximate Brodmann’s area; Hem, hemisphere; L, left; R, right; MNI, coordinates referring to the Montreal Neurological Institute template brain included in the SPM5 software package; *t*_max_, maximum *t* statistic in the cluster; *k*, cluster size in voxels; n.s., not significant. Model 1, regression of PPI on trait anxiety; Model 2, regression of PPI on trait anxiety and performance; Model 3, regression of PPI on trait anxiety, performance, and intelligence.

## Discussion

In the present study, we investigated how the neural efficiency of cognitive processing during working memory maintenance and manipulation is influenced by differences in trait anxiety. Within the a-priori-defined region of interest—that is, bilateral DLPFC—high-anxious participants showed stronger activation increases in a region of right DLPFC for working memory manipulation, as opposed to maintenance, than did low-anxious participants. Additional whole-brain analyses revealed that the high-anxious also showed stronger activation increases in a region in the left IFS as well as stronger decreases in rACC. The fact that, at the same time, high- and low-anxious participants did not differ in performance effectiveness supports the assumption of less-efficient neural task processing in anxious individuals.

Crucially, the effects were observed only for the manipulation of working memory contents—not for the mere maintenance of information in working memory. While simple maintenance primarily requires the short-term storage of information, the goal-directed manipulation of this information places additional demands on the top-down control of attention to sequentially refocus attention on different objects within the memory set and to rearrange them in a goal-directed manner (Eysenck et al., 2007; Oberauer, 2010; Oberauer & Bialkova, 2009). Furthermore, the effects were observed in brain regions that have most consistently been associated with executive-control processes. In addition, the observation of differences in DLPFC connectivity in association with anxiety suggests an important role for functional integration between brain regions in determining neural efficiency.

### Task effects across all participants: Manipulation versus maintenance

Across participants—not taking into account individual differences in trait anxiety—we observed an increase in response times, error rates, brain activation, and functional connectivity for the manipulation as contrasted to the maintenance of working memory contents that was in accordance with previous reports (e.g., Champod & Petrides, 2007; D’Esposito et al., 1999; D’Esposito et al., 2000; Postle et al., 1999; Van Hecke et al., 2010; Wager & Smith, 2003).

### Reduced neural efficiency in anxious participants

While trait anxiety did not affect performance in the working memory task, it did show a positive correlation with an increase in the neural effort expended for manipulation as compared to maintenance in a subregion of the a-priori-defined region of interest—that is, in the midportion of right DLPFC. When we extended our analysis to the whole brain, an equivalent effect was observed in left IFS (situated inferior to and slightly deeper than the effect in right DLPFC) and in a region in the brainstem. The positive correlations between anxiety and the strength of task-related brain activation in DLPFC and IFS most directly support the prediction that higher levels of trait anxiety are associated with reduced neural efficiency. The fact that, for the same level of behavioral performance, higher-anxious individuals expended more neural effort on task processing renders their processing less efficient.

Our results are consistent with the transient effects of trait anxiety on brain activation during working memory performance in a broader network of cognitive-control regions reported by Fales et al. (2008). These authors observed a positive correlation between anxiety and working-memory-related activation of a cognitive-control network comprising bilateral DLPFC, while anxiety—as in our study—did not affect performance. Whereas the *n*-back paradigm used to study working-memory-related brain activation by Fales et al. did not allow for distinguishing between maintenance- and manipulation-related changes in the fMRI signal, our results suggest that anxiety specifically affects the component function of manipulation. This is consistent with the assumption that anxiety impairs neural efficiency specifically for those cognitive functions that require the executive control of attention (Eysenck et al., 2007).

In a broader sense, the present finding of stronger DLPFC activation in high-anxious participants is also consistent with studies reporting effects of trait anxiety on neural efficiency for different cognitive tasks (i.e., other than working memory) that also require the top-down control of attention. For inhibitory control during Stroop performance (Basten et al., 2011) and for task switching in an antisaccade paradigm (Ansari & Derakshan, 2011a), trait anxiety was also associated with stronger, supposedly compensatory activation—along with equal (Ansari & Derakshan, 2011a) or worse (Basten et al., 2011) performance effectiveness, both of which would indicate reduced neural efficiency. However, other studies have used tasks that require attentional control for the purpose of inhibition, where trait anxiety was associated with decreased, supposedly insufficient activation—along with equally accurate but slower performance (Ansari & Derakshan, 2011b; Bishop, 2009), which does not allow for an unequivocal interpretation with respect to neural efficiency (see the introduction).

As outlined above, Basten et al. (2011), using fMRI, found stronger activation of the right DLPFC in high-anxious participants during the exertion of inhibitory control in the Stroop task, along with impairments of performance accuracy—which also remained when statistically controlling for variation in performance accuracy. Ansari and Derakshan (2011a), using EEG to study preparation-related neural activity for a task requiring switches between pro- and antisaccades, reported that high-anxious participants showed increased levels of neural effort, indicated by greater contingent negative variation amplitudes at frontal cortical sites, while their switching performance was comparable to that of low-anxious participants. On the other hand, on the basis of an fMRI study, Bishop (2009) reported weaker activation of the left DLPFC for high-anxious participants during distractor inhibition in a letter search task, along with equally accurate but slower responses. Ansari and Derakshan (2011b), using EEG, reported weaker neural activity in high- than in low-anxious participants during the exertion of inhibitory control in an antisaccade task, indicated by lower event-related potential activity at frontocentral cortical sites. Also in this study, high- and low-anxious participants did not differ in performance accuracy, yet the high-anxious were slower in performing correct antisaccades.

Only recently has a discussion evolved within the framework of attentional control theory about the idea that whether high-anxious individuals show weaker (supposedly insufficient) or stronger (supposedly compensatory) neural activation in brain regions supporting cognitive control may depend on task demands, motivational factors, and the opportunity to prepare for task performance (Ansari & Derakshan, 2011b; Eysenck & Derakshan, 2010). Studies that systematically manipulate the attentional-control demands of tasks, the motivational states of participants, and the preparation allowed by the context are needed to determine the conditions under which trait anxiety is indeed associated with compensatory increases in neural effort—and, in constrast, when anxious individuals are not able or not motivated to mobilize additional resources. Note, however, that while there are heterogeneous findings with respect to the executive function of inhibition (see the preceding paragraph), so far, empirical evidence is consistent with respect to working memory, where both the study of Fales et al. (2008) and our present findings point to compensatory increases in neural effort and reduced neural efficiency in high-anxious individuals.

Apart from stronger activation increases in DLPFC and IFS, the high-anxious participants in the present study also showed stronger deactivation—that is, decreases in fMRI signal in rACC. This region, also referred to as the *affective subdivision* of the ACC (Bush, Luu, & Posner, 2000), is part of the so-called *task-negative* or *default-mode* network, a set of functionally connected brain regions that typically show a decrease in fMRI signal during the goal-directed processing of cognitive tasks (Drevets & Raichle, 1998; Raichle et al., 2001). The findings that deactivation of the default network increases with task difficulty (McKiernan, Kaufman, Kučera-Thompson, & Binder, 2003; Singh & Fawcett, 2008) and that the extent of this deactivation is positively related to performance on cognitive tasks (Eichele et al., 2008; Li, Yan, Bergquist, & Sinha, 2007; Weissman, Roberts, Visscher, & Woldorff, 2006) suggest that deactivation in the default network reflects cognitive effort expended on task processing, and that deactivation is necessary for successful task performance. Thus, we interpret our finding of stronger rACC deactivation in the high-anxious as reflecting greater effort expended on the suppression of the brain’s default activity in order to support task performance.

Notably, our observation of anxiety effects on deactivation in the default network also supports a finding reported by Fales et al. (2008). For their verbal three-back task, Fales et al. observed greater sustained deactivation for high- as compared to low-anxious participants in the default network as a whole. Taken together, both of our findings strongly support the view that, for attention-demanding cognitive tasks, anxiety modulates both the up-regulation of cognitive control (associated with increased activity in the task-positive network) and the down-regulation of default-mode processes (associated with decreased activity in the task-negative, or default-mode, network). In as far as both task-related up- and down-regulation plausibly reflect cognitive effort, both support the prediction derived from attentional control theory (Eysenck et al., 2007) that anxiety is associated with greater neural effort—and thus with reduced neural efficiency.

### Enhanced functional connectivity in anxious participants

To further elucidate the characteristics of neural processing as depending on trait anxiety, in addition to differences in regional activation strength, we investigated interregional coupling within the task-relevant—that is, working memory manipulation specific—network. As generally cognitive processing is assumed to rely on neuronal communication among the different brain regions involved in task processing (Bressler, 1995; Bressler & Menon, 2010; Friston, 2002; Tononi, Edelman, & Sporns, 1998), it is reasonable to assume that the strength and nature of functional coupling within task-relevant networks in the brain codetermines the efficiency of cognitive processing. For the manipulation of working memory contents, high- as compared to low-anxious individuals showed stronger functional coupling of the right DLPFC seed region with regions in the right and—when adding intelligence as a predictor of no interest in the regression model—left VLPFC. Furthermore, enhanced coupling was observed with superior parts of the left and right cerebellum. A separate analysis using rACC as the seed revealed stronger coupling to adjacent parts of the rACC and to a region enclosing the central sulcus. In particular, for the right DLPFC, functional connectivity could reflect inhibitory or excitatory influences of a control region on the slave systems of working memory (Baddeley, 1986). Although, due to the correlational nature of the PPI analysis (see Friston et al., 1997), the present work does not support conclusions on the direction of the influences between regions showing a correlation in activation, in the present task context it is highly plausible that DLPFC connectivity would reflect top-down effects.

The enhanced connectivity observed in high-anxious participants may be disadvantageous, and thereby constitute a potential cause for compensatory activation increases in the right DLPFC. This interpretation would be in line with the interpretation adopted in our previous study on anxiety and alterations in functional connectivity during inhibitory control in the Stroop task, where the weaker connectivity shown by high-anxious individuals was interpreted as suboptimal (see Basten et al., 2011). Yet the enhanced connectivity observed in the present study could as well be advantageous, reflecting a compensatory increase in network connectivity in the high-anxious, enabling these participants to avoid detriments of performance, possibly triggered by enhanced control-related activation in DLPFC.

In activation studies, the VLPFC has been associated with the maintenance of information in working memory (D’Esposito et al., 1999; D’Esposito et al., 2000; Petrides, 2005; Postle et al., 1999), with the shielding of contents in working memory by inhibiting distracting information (Dolcos, Kragel, Wang, & McCarthy, 2006; Dolcos, Miller, Kragel, Jha, & McCarthy, 2007; Jha, Fabian, & Aguirre, 2004), and, more generally, with inhibitory processes supporting cognitive and action control (where, in particular, the right VLPFC has been associated with response inhibition; Aron, Robbins, & Poldrack, 2004). Cerebellar activation during verbal working memory tasks has predominantly been attributed to subvocal articulatory rehearsal processes (Ackermann, Mathiak, & Riecker, 2007; Ben-Yehudah & Fiez, 2008; Ben-Yehudah, Guediche, & Fiez, 2007; Chiricozzi, Clausi, Molinari, & Leggio, 2008; Hayter, Langdon, & Ramnani, 2007; Stoodley & Schmahmann, 2009). More specifically, activation in lateral portions of the superior cerebellum—the region displaying higher functional connectivity with our anxiety-modulated DLPFC region—has been implicated in covert speech, a strategy that is important for maintenance of verbal information, but very plausibly also contributes to working memory manipulation (Durisko & Fiez, 2010; Marvel & Desmond, 2010).

From the functions assigned to VLPFC and to lateral parts of the superior cerebellum in verbal working memory, it can be speculated that higher functional coupling of the DLPFC with bilateral VLPFC and cerebellum reflects stronger control of maintenance and rehearsal of information held in working memory. From previous research on individual differences in functional connectivity, we know that more connectivity is not always better: It depends on the task at hand and the brain regions involved, whether or not a particular increase or decrease in connectivity will be beneficial. In some cases, an increase in connectivity (coupling) has been beneficial, in the sense that it was associated with better performance (Meda, Stevens, Folley, Calhoun, & Pearlson, 2009; Neubauer & Fink, 2009b; Schlösser et al., 2003; Spoletini et al., 2009); in other cases, a decrease in connectivity (decoupling) was associated with better behavioral outcomes (Meyer-Lindenberg et al., 2005; Rypma et al., 2006; Stelzel, Basten, Montag, Reuter, & Fiebach, 2010). As in the present study the differences in connectivity were not associated with differences in behavioral performance measures (for correlations between PPI estimates and performance, see Table S6 in the supplementary materials), we cannot conclusively decide whether the observed alterations in functional connectivity are of a compensatory nature and support task performance, or whether they are dysfunctional and thus hinder task performance. Future research must strive to elucidate the specific functional significance of differences in the strength of functional connectivity between DLPFC and VLPFC as well as cerebellum in the context of working memory manipulation—for instance, by parametrically increasing task difficulty and analyzing the effects on functional connectivity in association with behavioral performance.

Notwithstanding the need to further investigate the functional significance of the observed differences in connectivity, the present findings underscore our hypothesis that the quality of functional integration of distributed, task-relevant brain networks varies between individuals and should be considered as a variable codetermining neural efficiency when studying the effects of trait anxiety on cognitive processing (cf. Basten et al., 2011). Alterations in functional connectivity may provide the key for understanding the reductions in processing efficiency observable in high-anxious participants.

### Conclusions

The present study has provided most stringent support for the assumptions of attentional control theory—that in tasks requiring attentional control, anxiety impairs processing efficiency more than performance effectiveness (cf. Eysenck et al., 2007). In the working memory manipulation task that we investigated, anxiety did not affect behavioral performance, yet it was positively associated with task-related activation increases in regions centrally involved in cognitive control—that is, right DLPFC and left IFS—and with decreases in a region of the default-mode network—that is, rACC. We interpret both results as reflecting the reduced neural efficiency of attentional-control processes in high-anxious participants. For effective compensation of a general deficit in the goal-directed control of attention, the down-regulation of default-mode processes may be just as important as the up-regulation of cognitive control (see also Eichele et al., 2008; Fales et al., 2008; Li et al., 2007; Weissman et al., 2006). Anxiety also was associated with a stronger coupling of right DLPFC with bilateral VLPFC and superior cerebellum. The finding of anxiety-dependent alterations in the functional coupling of distributed task-related networks is in line with previous reports (Basten et al., 2011) and demonstrates the importance of considering measures of functional integration in combination with measures of regional activation strength when investigating individual differences in neural processing efficiency.

## Electronic supplementary material

Below is the link to the electronic supplementary material.

ESM 1 (PDF 478 kb)
