# Supplementary material for: Trait anxiety and the neural efficiency of manipulation in working memory
Source: Cogn Affect Behav Neurosci. 2012 May 29;12(3):571–88. doi: 10.3758/s13415-012-0100-3 (PMC3400031; doi:10.3758/s13415-012-0100-3)
Supplement: Supplementary file 1 — (PDF 478 kb) [file 13415_2012_100_MOESM1_ESM.pdf]

SUPPLEMENTARY MATERIAL

Trait Anxiety and the Neural Efficiency of Manipulation in Working Memory

Ulrike Basten, Christine Stelzel, Christian J. Fiebach

Content

Supplementary Results

Brain Activation During Encoding and Retrieval

Effects of Trait Anxiety on Brain Activation During Encoding and Retrieval

Negative Effects of Task on Brain Activation During the Task Delay

Supplementary Tables

Table S.1: Brain Activation During Encoding and Retrieval

Table S.2: Effects of Trait Anxiety on Brain Activation During Retrieval

Table S.3: Negative Effects of Task on Brain Activation During the Task Delay

Table S.4: Functional Connectivity of Left IFS and rACC

Table S.5: Increased Functional Connectivity with rACC in High Anxious

Table S.6: Correlations of Performance measures with PPI estimates

Supplementary Figures

Figure S.1: Brain Activation During Encoding and Retrieval

Figure S.2: Effects of Trait Anxiety on Brain Activation During Retrieval

Figure S.3: Negative Effects of Task on Brain Activation During the Task Delay

SUPPLEMENTARY RESULTS

Brain Activation During the Encoding and the Retrieval Period

This paragraph summarizes task-related brain activation observed across all participants, i.e., not taking into account differences in trait anxiety. During the encoding period, which did not differ between the two conditions investigated (i.e., maintenance and manipulation), increased brain activation ([encoding_maintenance + encoding_manipulation] > baseline) was observed in ventrolateral prefrontal cortex (VLPFC), pre- and postcentral sulcus, intraparietal sulcus (IPS), supplementary motor areas (SMA), preSMA and dorsal anterior cingulate cortex (dACC), thalamus, globus pallidus and putamen, middle temporal gyrus (MTG), occipital lobe, and in the cerebellum (Figure S.1A, Table S.1A). During the retrieval period, stronger brain activation was observed following the manipulation of working memory contents than following the mere maintenance (retrieval_manipulation > retrieval_maintenance). Clusters of activation were situated bilaterally in the middle frontal gyrus, extending into superior frontal gyrus, bilaterally in the intraparietal sulcus and adjacent inferior and superior parietal cortex, and in medial parts of the precuneus (Figure S.1B, Table S.1B). Brain activation for the task delay period of the task is reported in the main text.

Effects of Trait Anxiety on Brain Activation During the Encoding and Retrieval Period

As during the encoding period, i.e., prior to the presentation of the task cue, the two task conditions we investigated did not differ, here we could not test for the interaction of task condition and anxiety – which was the primary focus of the current study (see Introduction, main text). Nevertheless, we tested for anxiety effects also during the encoding period, to test whether or not high- and low-anxious individuals did differ with respect to brain activation in task periods preceding the one we focused our analyses on. Anxiety showed no effect on encoding-related brain activation ([encoding.maintenance + encoding.manipulation] >

baseline), neither when testing within the DLPFC ROI nor when testing at the whole-brain level. Also during the retrieval period trait anxiety did not significantly affect task-related brain activation in our primary region of interest, i.e., in bilateral DLPFC. However, in the analysis considering the whole brain, an interaction of anxiety and task condition on activation for manipulation as contrasted to maintenance was observed in an extensive cluster located alongside the intraparietal sulcus, including effects in bilateral superior and inferior parietal gyri and precuneus. In addition to the parietal effects, the interaction was also observed in antero-medial PFC (AMPFC), precentral gyrus, posterior cingulate cortex, temporal lobe, fusiform gyrus, occipital lobe, and in the cerebellum (Table S.2, Figure S.2A).

Scatterplots illustrate that the effects were not driven by outliers (Figure S.2B, exemplarily for left and right parietal lobe and AMPFC. Notably, plotting of the beta estimates (Figure S.2C, exemplarily for the same three regions) revealed a different pattern of interaction between condition and anxiety group than observed during the task-delay period (see main text). In all regions showing a significant interaction during the retrieval period,
the high-anxious group showed stronger activation for retrieval after manipulation in comparison to retrieval after maintenance, whereas the low-anxious group showed the opposite pattern, i.e., stronger activation for retrieval after maintenance than for retrieval after manipulation.

This crossover interaction pattern cannot simply be explained in terms of more or less neural effort invested by either group. Future research should aim at elucidating how cognitive activity during the delay period of working memory tasks influences representations available in the retrieval period and thus retrieval-related brain activation. For the time being, we can only speculate that for high- vs. low-anxious individuals, working memory representation at the end of the task delay phase were differentially stable or accessible, possibly due to

different degrees of pre-activation. This could explain why we observed not only quantitatively but qualitatively different activation patterns for high- as compared to low-anxious individuals.

Negative Effects of Task on Brain Activation During the Task Delay Period

During the task delay period negative effects of task processing on brain activation (baseline > taskdelay_maintenance + taskdelay_manipulation) were observed in a set of regions commonly assigned to what has been called the task-negative or default mode network of the brain (Raichle et al., 2001, Table S.3, Figure S.3). In our task, reduced activation during task-processing as compared to baseline was observed in the medial frontal gyrus including the rostral-ventral part of the anterior cingulate cortex (rACC), in the superior frontal gyrus, the posterior cingulate cortex, precuneus and cuneus, in the area of the temporo-parietal junction, and in the middle and inferior temporal gyrus.

SUPPLEMENTARY TABLES

Table S.1

Brain Activation During the Encoding and the Retrieval Period

Brain region	BA	Hem	MNI			T_{max}	k
			x	*y*	*z*		
**(A) Encoding Period** (Maintenance + Manipulation > Baseline) *Voxel height threshold T = 5.33, Cluster extend threshold: k = 27 voxels*							
Precentral and postcentral gyrus, intraparietal sulcus (IPS), ventrolateral prefrontal cortex (VLPFC)	1/2/3/4/6/7/40/44	L	-56	-8	42	13.19	4768
Precentral and postcentral gyrus, intraparietal sulcus (IPS)	2/3/4/6/7/40	R	48	-30	44	9.29	1668
Supplementary motor area (SMA), presupplementary motor area (preSMA), anterior cingulated cortex (ACC)	6/24/32	R/L	-4	4	54	14.35	2154
Thalamus, globus pallidus, putamen		L	-16	2	2	8.17	703
Middle temporal gyrus (MTG)	21	L	-58	-28	-4	6.66	82
Occipital lobe	18/19	R	32	-92	6	12.48	761
	18/19	L	-26	-96	-2	10.01	438
Cerebellum		R	24	-68	-24	10.29	1400
		R	16	-66	-42	7.74	74
		L	-22	-66	-24	6.31	62
**(B) Retrieval Period** (Manipulation > Maintenance) *Voxel height threshold T = 5.48, Cluster extend threshold: k = 23 voxels*							
Middle frontal gyrus (MFG), superior frontal gyrus (SFG)	8	R	34	24	50	8.14	363
		L	-36	18	52	7.80	610
Intraparietal sulcus (IPS), inferior and superior parietal lobule	7/39/40	R	58	-58	36	11.05	1021
		L	-46	-56	32	10.30	1152

Precuneus	31	R/L	0	-54	38	6.58	87

Note. BA: approximate Brodmann's area; Hem: hemisphere, L: left, R: right; MNI: coordinates referring to the Montreal Neurological Institute template brain included in the SPM5 software package; T_{max}: maximum t-statistic in the cluster; *k*: cluster size in voxels. Activation is reported for a voxel-level threshold of $p < .05$ and a cluster-level threshold of $p < .001$, both FWE corrected.

Table S.2

Effects of Trait Anxiety on Brain Activation (Manipulation > Maintenance) for the Retrieval Period

			MNI*			Model 1		Model 2		Model 3	
Brain region	BA	Hem	*x*	*y*	*z*	T_{max}	*k*	T_{max}	*k*	T_{max}	*k*
**Positive Correlation**<br>*Voxel height threshold T = 2.70, Cluster extend threshold: k = 142 voxels*											
Antero-medial PFC	10	R/L	-4	62	14	4.08	254	4.06	220	3.85	156
Precentral gyrus	6	R	28	-20	54	3.60	148	3.64	147	3.55	n.s. (92)
Posterior cingulate cortex, middle temporal gyrus, transverse temporal gyrus	23/30	L	-38	-50	8	4.31	462	4.59	476	4.70	1009
Posterior cingulate gyrus	23	R	24	-54	26	4.14	239	4.13	203	"	"
Transverse temporal gyrus	41	R	32	-36	12	4.20	184	4.25	174	"	"
Middle temporal gyrus	21	L	-40	-16	-18	4.21	342	4.15	325	4.25	407
Intraparietal sulcus, precuneus	7/39/40	R	32	-76	36	5.02	3565	4.93	1678	4.72	1337
	7/19/39	L	-14	-74	50	"	"	4.15	961	4.14	864
Intraparietal sulcus	5/7/40	L	-14	-46	56	"	"	4.21	513	3.99	267
Fusiform gyrus	20/37	L	-40	-46	-22	4.68	346	4.69	354	4.55	365
Occipital lobe	18/19/37	R	48	-66	0	3.69	764	3.59	689	3.42	396
Cerebellum		R/L	0	-78	-34	3.94	n.s. (136)	3.87	142	3.94	164

Note. BA: approximate Brodmann's area; Hem: hemisphere, L: left, R: right; MNI: coordinates referring to the Montreal Neurological Institute template brain included in the SPM5 software package; *MNI coordinates are reported for peaks from model 2, coordinates for model 1 and 3 lay in the same cluster; T_{max}: maximum t-statistic in the cluster; *k*: cluster size in voxels; n.s.: not significant; ": peak fell within the cluster specified in the row above. Model 1: Regression of PPI on trait anxiety. Model 2: Regression of PPI on trait anxiety and performance. Model 3: Regression of PPI on trait anxiety, performance, and intelligence.

Table S.3

Negative Effects of Task on Brain Activation During the Task Delay Period

Brain region	BA	Hem	MNI *x*	MNI *y*	MNI *z*	T_{max}	*k*
**Task Delay Period** (Baseline > Maintenance + Manipulation)<br>*Voxel height threshold T = 5.27, Cluster extend threshold: k = 49 voxels*							
Medial frontal gyrus, (rostral part of) anterior cingulate cortex (ACC), superior frontal gyrus (SFG)	9/10/32	R/L	4	44	-14	12.45	5175
Posterior cingulate cortex, precuneus	23/30/31	R/L	-2	-48	66	9.98	2777
Precuneus, cuneus	7/19	R/L	18	-82	44	9.54	1319
Temporo-parietal junction area (TPJ), posterior parts of middle and inferior temporal gyrus	22/37/39/40	R	58	-56	28	9.14	1780
Temporo-parietal junction area (TPJ)	39/40	L	-56	-60	34	8.96	555
Middle temporal gyrus	21	R	64	-6	-14	7.84	429
		L	-60	-8	-22	6.78	248
Temporal pole (superior temporal gyrus, near amygdala)	38	R	32	6	-24	6.26	54
Fusiform gyrus, parahippocampal gyrus	19/20/37	L	-30	-38	-14	7.39	526

Note. BA: approximate Brodmann's area; Hem: hemisphere, L: left, R: right; MNI: coordinates referring to the Montreal Neurological Institute template brain included in the SPM5 software package; T_{max}: maximum t-statistic in the cluster; *k*: cluster size in voxels. Activation is reported for a voxel-level threshold of $p < .05$ and a cluster-level threshold of $p < .001$, both FWE corrected.

Table S.4

Functional Connectivity of Left IFS and rACC Seed Regions During Working Memory Manipulation

Brain region	BA	Hem	MNI x	y	z	T_{max}	k
**Seed: Left inferior frontal sulcus (IFS), Task Delay Phase** (Manipulation > Maintenance) *Voxel height threshold T = 2.70, Cluster extend threshold: k = 142 voxels*							
Occipital Lobe	17/18	L/R	-12	-96	2	5.15	568
**Seed: Rostral-ventral anterior cingulate cortex (rACC), Task Delay Phase** (Manipulation > Maintenance) *Voxel height threshold T = 2.70, Cluster extend threshold: k = 142 voxels*							
Inferior parietal lobe, precentral gyrus, postcentral gyrus, superior and middle temporal gyrus, posterior cingulate gyrus, posterior parts of middle frontal gyrus	1/2/3/4/6/7/ 21/22/23/37/ 39/40/41/42	R	64	-34	30	6.28	7174
Inferior parietal lobe, precentral and postcentral gyrus, superior and middle temporal gyrus,	1/2/3/4/22/37/ 39/40/41/42	L	-30	-72	8	5.55	4166
Parietal lobe, precuneus	7/39	R	32	-72	28	4.44	628
Middle temporal gyrus	21	R	62	-28	-6	4.31	388
Caudate Head		L	-20	22	10	4.23	152

Note. BA: approximate Brodmann's area; Hem: hemisphere, L: left, R: right; MNI: coordinates referring to the Montreal Neurological Institute template brain included in the SPM5 software package; T_{max}: maximum t-statistic in the cluster; k: cluster size in voxels.

Table S.5

Regions Showing Increased Functional Connectivity With the rACC Seed Region in High Anxious Subjects During Working Memory Manipulation

			MNI			Model 1		Model 2		Model 3	
Brain region	BA	Hem	*x*	*y*	*z*	T_{max}	*k*	T_{max}	*k*	T_{max}	*k*
**Positive Correlation**											
Voxel height threshold T = 2.70, Cluster extend threshold: k = 142 voxels											
Rostral-ventral anterior cingulate cortex (rACC), orbitofrontal cortex (OFC)	11/12/ 32/47	L	-10	36	0	4.34	222	4.25	235	4.50	239
		R	26	18	-14	4.31	326	4.24	369	4.16	359
Pre- and postcentral gyrus	3/4	L	-62	-14	26	4.09	271	4.20	251	4.07	190

Note. BA: approximate Brodmann's area; Hem: hemisphere, L: left, R: right; MNI: coordinates referring to the Montreal Neurological Institute template brain included in the SPM5 software package; T_{max}: maximum t-statistic in the cluster; *k*: cluster size in voxels; Model 1: Regression of PPI on trait anxiety. Model 2: Regression of PPI on trait anxiety and performance. Model 3: Regression of PPI on trait anxiety, performance, and intelligence. Reported are all clusters that showed a significant effect in all three models tested.

Table S.6

Correlations of Performance measures with PPI estimates for the Working-Memory-Manipulation Task

Condition	PM	Right VLPFC		Left VLPFC		Right Cerebellum		Left Cerebellum	
Overall	ERR	.18	(.222)	.16	(.301)	-.01	(.971)	-.01	(.930)
	RT	-.03	(.842)	-.00	(.992)	-.13	(.392)	-.20	(.175)
Maintenance (MA)	ERR	.15	(.329)	.15	(.320)	.07	(.642)	-.01	(.934)
	RT	-.00	(.983)	.05	(.757)	-.10	(.513)	-.14	(.339)
Manipulation (MP)	ERR	.16	(.294)	.12	(.434)	-.05	(.729)	-.01	(.946)
	RT	-.06	(.718)	-.05	(.747)	-.15	(.319)	-.25	(.097)
Difference (MP - MA)	ERR	.07	(.660)	.02	(.877)	-.10	(.505)	-.00	(.988)
	RT	-.10	(.512)	-.18	(.223)	-.10	(.500)	-.21	(.172)

Note. p-values are given in brackets. PM: performance measure, ERR: error rate, RT: response time. VLPFC: ventrolateral prefrontal cortex. PPI estimates were extracted for the brain regions showing a significant modulation of functional connectivity with the right DLPFC seed region by trait anxiety.

References

Raichle, M. E., MacLeod, A. M., Snyder, A. Z., Powers, W. J., Gusnard, D. A., & Shulman, G. L. (2001). A default mode of brain function. *Proc Natl Acad Sci U S A, 98*, 676-682.

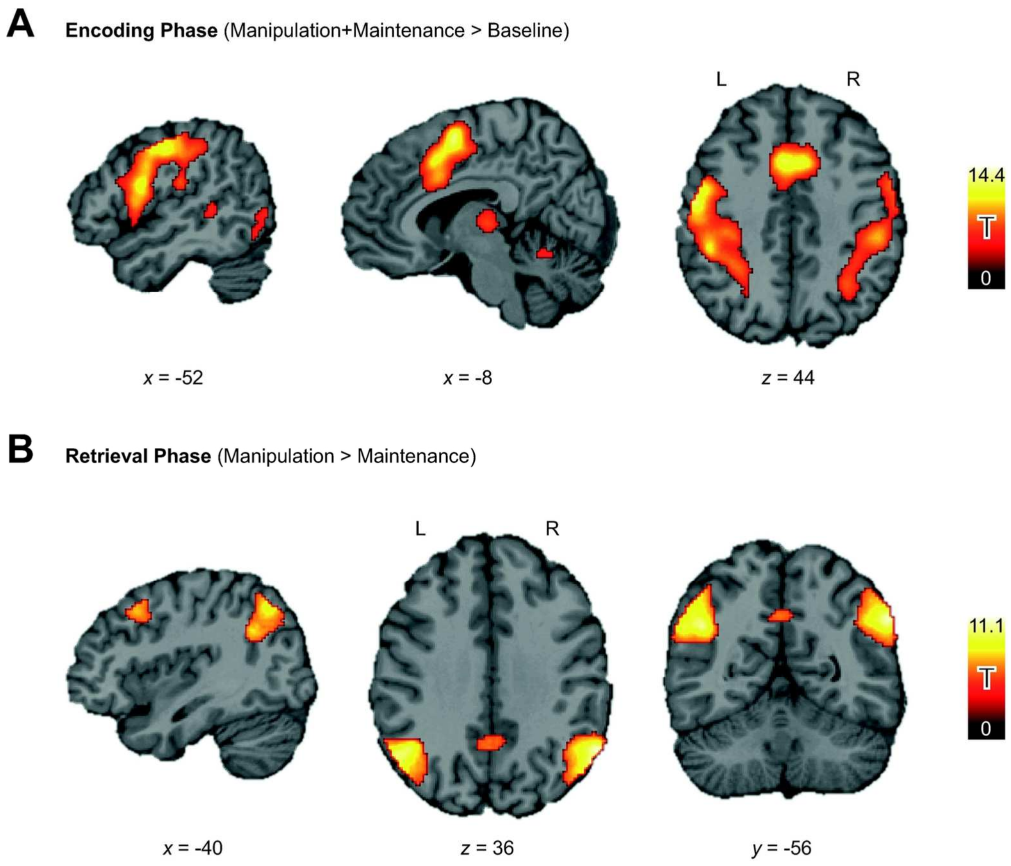

Figure S.1: Brain activation for working memory encoding (A) and retrieval (B). Activation is illustrated at a voxel-level threshold of p < .05 and a cluster-level threshold of p < .001, both FWE corrected. L: left; R: right; x, y, z : coordinates referring to the Montreal Neurological Institute template brain included in the SPM5 software package. DACC: dorsal anterior cingulate cortex; IPS: intraparietal sulcus; MFG: middle frontal gyrus; MTG: middle temporal gyrus; preSMA: presupplementary motor area; SMA: supplementary motor area; VLPFC: ventrolateral prefrontal cortex.
140x121mm (300 x 300 DPI)

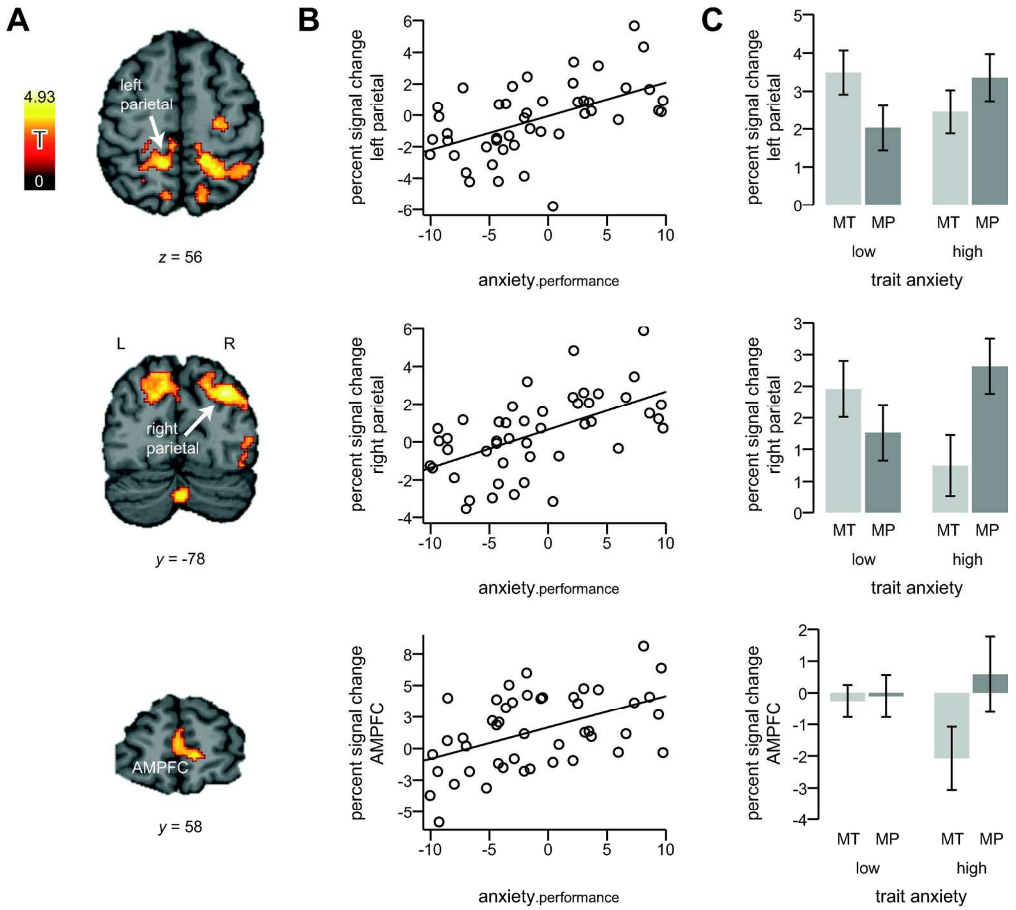

Figure S.2: Effects of trait anxiety on brain activation during the retrieval period of the working memory task – controlling for (non-significant) variation in performance. (A) Statistical parametric maps are shown at a voxel-level threshold of $p < .005$. L: left; R: right; y, z: coordinates referring to the Montreal Neurological Institute template brain included in the SPM5 software package. Arrows indicate the region for which effects are plotted in the respective row of B and C. (B, C) Percent signal change extracted from the regions indicated in A. (B) Percent signal change for manipulation > maintenance plotted against anxiety.performance: residual of trait anxiety from regression on behavioral performance. (C) Comparison of mean percent signal change for task (MP: manipulation > zero, dark grey) and reference condition (MT: maintenance > zero, light grey) by trait anxiety group (median split). Error bars show the standard error of the mean.
178x161mm (300 x 300 DPI)

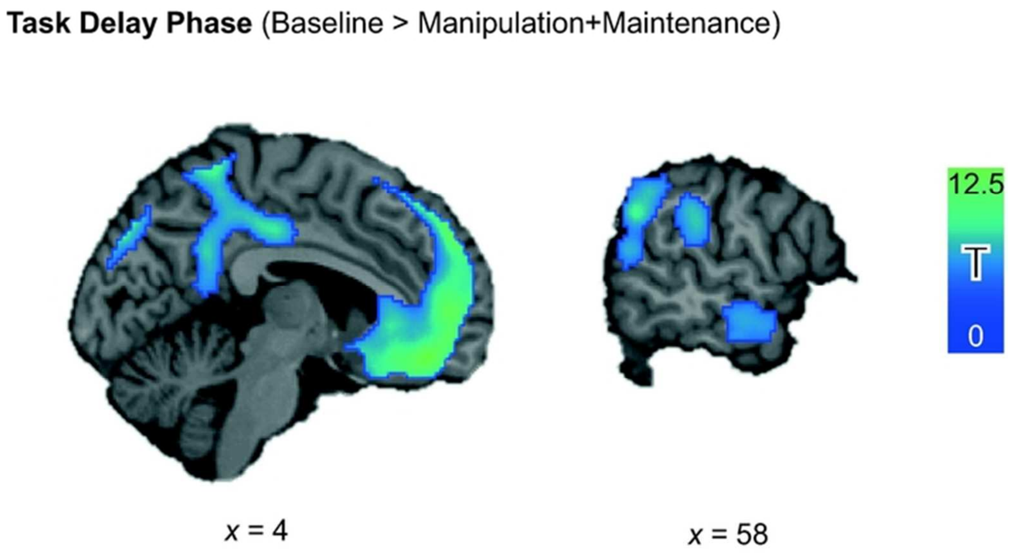

Figure S.3: Task-negative network: Brain de-activation during the task delay period of the working memory task (baseline > maintenance + manipulation). Activation is illustrated at a voxel-level threshold of p < .05 and a cluster-level threshold of p < .001, both FWE corrected. x: coordinates referring to the Montreal Neurological Institute template brain included in the SPM5 software package. AMPFC: anteromedial prefrontal cortex; MTG: middle temporal gyrus; PCC: posterior cingulate cortex; rACC: rostral anterior cingulate cortex; SFG: superior frontal gyrus; TPJ: temporo-parietal junction area.
59x33mm (300 x 300 DPI)
